# Case Report: Neurofilament light chain in the follow up of progressive multifocal leukoencephalopathy in a patient with multiple sclerosis treated with ocrelizumab

**DOI:** 10.3389/fphar.2025.1571699

**Published:** 2025-04-09

**Authors:** Raquel Piñar-Morales, Raquel Calle-Calle, Maria Carrasco-Garcia, Cristina Davila-Arias, Luisa Maria Villar-Guimerans, Francisco J. Barrero Hernandez

**Affiliations:** ^1^ Servicio de Neurología, Hospital Universitario San Cecilio, Granada, Spain; ^2^ Instituto de Investigación Biosanitaria ibs.GRANADA, Universidad de Granada, Granada, Spain; ^3^ Servicio de Radiología, Hospital Universitario San Cecilio, Granada, Spain; ^4^ Servicio de Inmunología, Hospital Universitario Ramón y Cajal, Madrid, Spain; ^5^ Departamento de Medicina, Facultad de Medicina, Universidad de Granada, Granada, Spain

**Keywords:** John Cunningham virus, multiple sclerosis, neurofilament light chain, ocrelizumab, progressive multifocal leukoencephalopathy

## Abstract

Progressive multifocal leukoencephalopathy (PML) results from the reactivation of John Cunningham virus JC virus and is a rare complication of anti-CD20 drug therapy. Neurofilament light chains increasingly serve as a marker of neuroaxonal damage in the follow-up of patients with multiple sclerosis (MS), but its role in the monitoring and detection of processes such as PML is yet to be defined. We report the case of a patient with MS who was treated with ocrelizumab and developed PML. Results: Serum neurofilament light chain (sNFL) levels were elevated at the diagnosis and progressively increased over his follow-up. Our results suggest that the monitoring of sNFL levels may be useful for the early diagnosis of PML in patients with MS.

## 1 Introduction

Progressive multifocal leukoencephalopathy (PML) is a rare and often lethal disease caused by reactivation of the John Cunningham virus JC virus in immunocompromised patients (Schweitzer et al., 2023). JC virus is a highly ubiquitous polyomavirus that remains latent in a large proportion of healthy individuals (Bozic et al., 2014). It was first described in individuals with hematological diseases and then in people living with human immunodeficiency virus (HIV). It has recently been associated with the receipt of immunosuppressive drugs and with immune system deficiencies that favor virus reactivation (Cortese et al., 2021). One of the most frequently implicated drugs is natalizumab, which acts against alpha-4 integrin and is used in the treatment of multiple sclerosis (MS). PML has also been related to other disease-modifying therapies (DMTs) for MS, such as fingolimod or dimethyl fumarate. An association between PML and ocrelizumab (OCR) has only been described in two patients (Rindi et al., 2024). Neurofilaments light chain (NfL) are key indicators of axonal damage and hold great promise as biomarkers for monitoring patients with MS. In the case of patients with PML, their measurement has been reported in various publications, although their role in the early detection of PML remains to be defined. We report the case of a patient with progressive primary MS (PPMS) who was treated with OCR and developed PML. Since the diagnosis of PML and during follow-up, serum neurofilament light chain (sNfL) has been determined.

## 2 Case presentation

**FIGURE 1:**
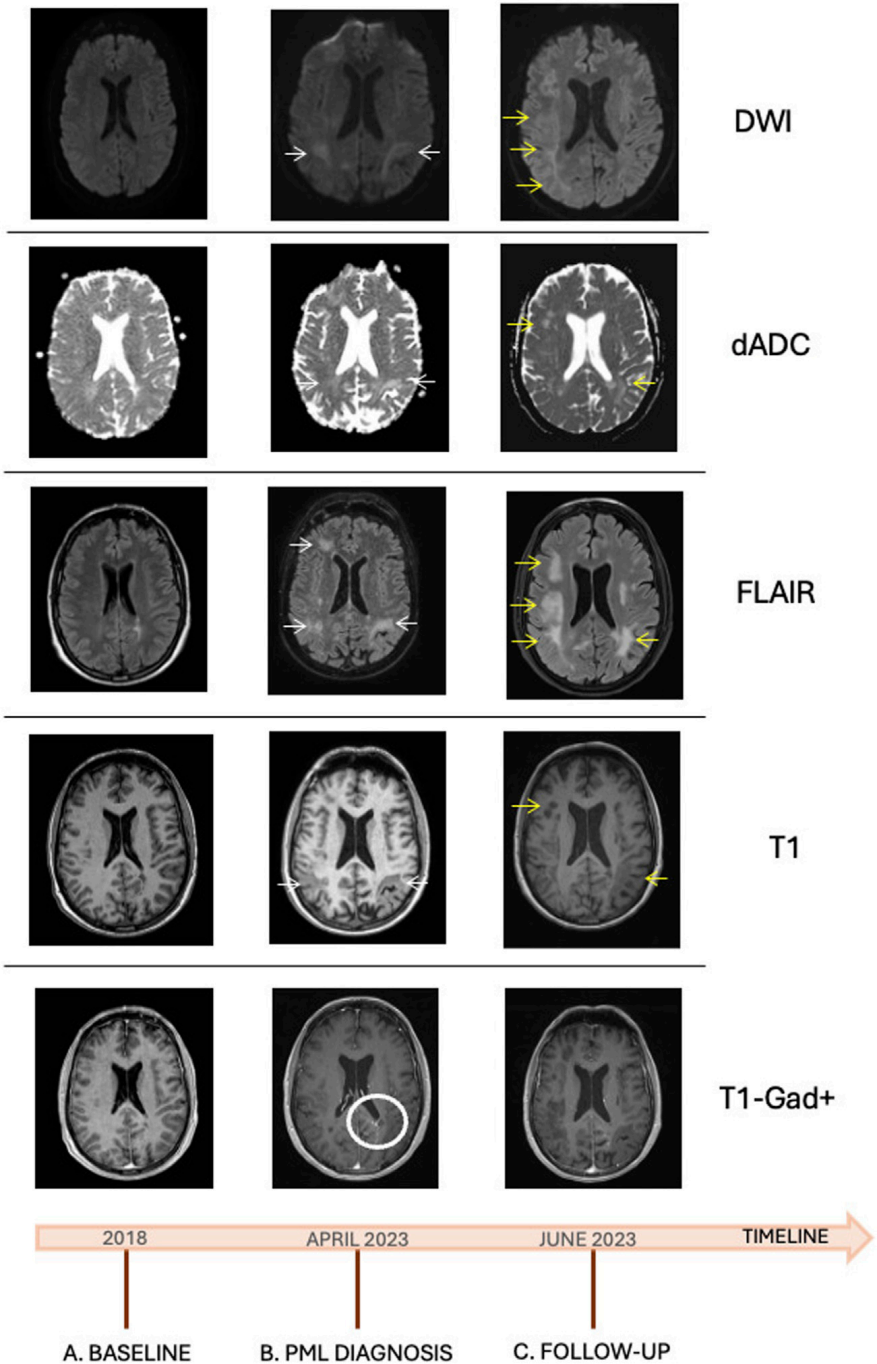
Neuroimaging study during follow up: **(A)** baseline MRI, 6 months before OCR treatment onset, observing hyperintense T2/FLAIR lesions with no contrast enhancement or diffusion restriction; **(B)** MRI performed upon PML diagnosis, observing subcortical lesions and lesions in both parietal and occipital lobes, T1 hypointense and FLAIR hyperintense (white arrows), with no expansive effect, contrast enhancement, or diffusion restriction. Contrast enhancement in the left parieto-temporal region related to a venous development anomaly, previously known (white circle) **(C)** Follow-up MRI after symptom worsening, observing an expansion of PML lesions (yellow arrows), with no contrast enhancement or diffusion restriction. dADC, Apparent diffusion coefficient ADC map; DWI, Diffusion-weighed imaging; FLAIR, fluid-attenuated inversion recovery; PML, progressive multifocal leukoencephalopathy; OCR, ocrelizumab; MRI, magnetic resonance imaging; T1-Gad+, T1 sequence with gadolinium.

56-year-old right-handed male diagnosed with PPMS (2010 McDonald criteria) in 2013. He had a history of latent tuberculosis and pulmonary nodule lesion, with a pathological diagnosis of necrotizing granuloma and a positive PCR result for *Mycobacterium tuberculosis*, and he underwent a complete chemoprophylaxis cycle in 2017. At the PPMS diagnosis, the patient reported weakness and gait abnormality during the previous year, and examination revealed proximal and distal weakness of 4/5 in right lower limb alongside pyramidal syndrome with bilateral Babinski sign. Neuroimaging showed spatial dissemination of demyelinating lesions and IgG- and IgM-positive oligoclonal bands in the cerebrospinal fluid (CSF). The expanded disability status scale (EDSS) score at diagnosis was 3.5. The patient was treated with glatiramer acetate (GA) for transient improvement after corticosteroid bolus administration. Follow-up neuroimaging study in 2018 evidenced radiological activity, with the emergence of new lesions (Figure 1A). In February 2019, the GA regimen was interrupted to initiate treatment with OCR, which was well tolerated. At OCR treatment onset, his EDSS score was 6.5, Timed 25-foot Walk Test (T25FW) was 18.25 s, 9-hole peg test (9HPT) 21.7 s in dominant hand (D) and 24.73 s in non-dominant hand (ND), immunoglobulin IgG was 937 mg/dL (normal range 540–1882 mg/dL) and IgM 513 mg/dL (normal range 22–240 mg/dL). A slight initial improvement was followed by a gradually progressive deterioration. At follow-up examination in December 2022, the 9HTP result was 25.10 s in D and 27.20 s in ND, the EDSS score remained stable, and there was no significant change in T25FW score (16.9 s) or IgG value (989 mg/dL), while IgM levels had progressively decreased but remained within the normal range (44 mg/dL). He received OCR treatment for 4 years, with no infusion-related adverse effects and no onset of severe infection.

In April 2023, he reported language problems over the previous 3 months, with confusion of phonemes and difficulty to find words, being able to understand what he read but unable to remember it immediately afterwards. The most recent OCR dose had been administered in December 2022. Examination revealed low-fluency spontaneous language with frequent blocks and some phonemic paraphasia. He understood simple orders but evidenced saturation with more complex orders, naming was preserved, and the result of the categorical verbal fluency test was six animals in 1 minute. He committed errors in reading with preserved writing. The cranial nerves were preserved. He had minimal distal weakness in right upper limb and paraparesis with right lower limb predominance. He had preserved symmetrical tactile sensitivity and algesia, and lower limb hypoesthesia.

Magnetic resonance imaging (MRI) (Figure 1B) showed multiple subcortical lesions and lesions in both parietal and occipital lobes, hypointense in T1 and hyperintense in T2/FLAIR, with no expansive effect, contrast uptake, or diffusion restriction. In addition, lesions were observed in the frontal lobe of the left hemisphere, which would explain the language impairment. Hyperintense supra- and infra-tentorial lesions in T2 were related to the presence of MS. Findings of no restriction in diffusion and no contrast uptake were compatible with PML. Blood count and immunoglobulin test results were within normal ranges, with IgA of 197 mg/dL (normal range, 70–400 mg/dL), IgG of 989 mg/dL (540–1882 mg/dL), and IgM of 45 (22–240 mg/dL). Lymphocyte counts were 1/μL for CD19 (normal range, 122–682/μL), 812/μL for CD4^+^ (540–1,660/μL), and 655/μL for CD8^+^ (270–930/μL). HIV serology was negative, and remaining proteinogram, serology, and autoimmunity results were negative or normal. Anti-aquaporin-4 antibodies (AbAQP4), anti-myelin oligodendrocyte antibodies (antiMOG Ab), anti-glial fibrillary acidic protein antibodies, and antineuronal antibodies were negative. CSF analysis showing no cellularity and 23.3 mg/dL proteins with 60 mg/dl glucose. Immunophenotyping in CSF showed low cellularity. Serum neurofilament light chain (sNfL) levels were 43.3 pg/mL. PCR studies in CSF were negative for viruses and bacteria, including toxoplasma. The determination of JC virus in CSF by PCR was performed at the Virgen de las Nieves Hospital in Granada with a positive result.

**FIGURE 2:**
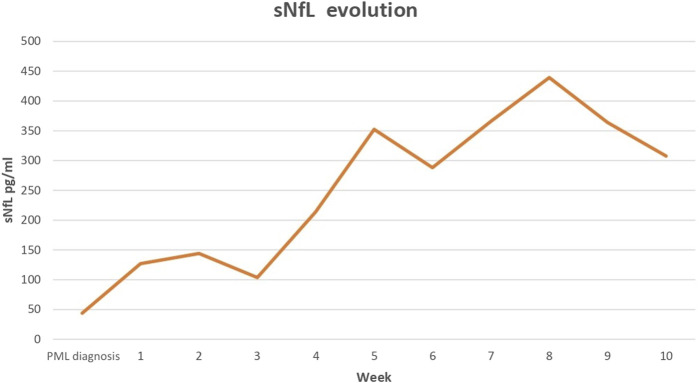
Time course of sNfL levels from PML diagnosis until death of patient. PML, progressive multifocal leukoencephalopathy; sNFL, serum neurofilament light chain.

Given the clinical and radiological characteristics, along with the positivity of JC virus in the CSF, he was diagnosed with definitive PML. Treatment with mirtazapine 30 mg daily and mefloquine 250 mg twice a day, later once weekly, was administered as compassionate treatments due to the absence of approved options. The patient remained clinically stable for the first 3–4 weeks, with no radiological worsening, and his sNfL levels were also followed at weekly examinations (Figure 2). Mild motor worsening was recorded in June 2023, and the patient was again admitted to hospital. An MRI scan evidenced larger and more extensive lesions (Figure 1C), and he died after a few days of clinical deterioration with increasing language disorder and motor impairment.

## 3 Discussion

The pathogenesis of PML has not yet been fully elucidated, and it is not known why this severe disease develops in certain patients. However, sustained immunosuppression plays a key role, given the high seroprevalence of JC virus in the general population. JC virus is ubiquitous and transmitted person-to-person or by contact with contaminated surfaces, food, or water (Cortese et al., 2021). The virus remains latent in kidneys, lymph nodes, and the brain, among other organs (Monaco et al., 1996). Chronic immunosuppression favors genetic changes that can foster neurotropism and brain damage by the virus (Cortese et al., 2021). In addition, virus clearance is hampered by the immunosuppression of lymphocytes, especially CD4^+^ and also CD8^+^ T lymphocytes, and by cytokine profile changes in the setting of immunosuppression (Bernard-Valnet et al., 2021).

PML has been described as a complication of anti-CD20 drug therapy since 2002, when it was first associated with rituximab treatment for patients with non-Hodgkin lymphoma (Bennett et al., 2021) (Calabrese et al., 2007). Most reported cases related to anti-CD20 drugs have involved patients receiving rituximab for MS, hematological neoplasms, or rheumatological diseases such as rheumatoid arthritis or sarcoidosis (Focosi et al., 2019). The mechanism by which these drugs favor virus reactivation and PML emergence is unknown but appears to involve factors related to the virus *per se* and to humoral and cellular immunity (Durali et al., 2015). It has been proposed that JC virus can remain as an intact virion after infecting B lymphocytes (BLs), which can therefore propagate and disseminate the virus in the central nervous system (CNS) (Chapagain and Nerurkar, 2010). Furthermore, JC virus DNA has been detected in peripheral BLs and BLs from PML-derived brain tissue (Major et al., 1990). Antibodies against VP1, the main protein of this virus, may play a neutralizing role and may be related to the number of activated plasmatic cells, although their protective effect remains unclear. In addition, given that BLs regulate T lymphocyte functionality via different pathways, BL depletion may alter homeostasis and facilitate virus persistence through a failure of effector T lymphocytes and inhibition of the inflammatory response (Durali et al., 2015).

JC virus reactivation with PML development is a highly infrequent complication of some DMTs in MS. To our best knowledge, we present only the third published case of PML associated with OCR in a patient not previously treated with another DMT. There have been reports of PML onset in seven patients who had previously received natalizumab or fingolimod (Sharma et al., 2022). The first reported case of a patient with PML treated with OCR and without prior DMT was a 78-year-old male who had received OCR for 2 years Lymphocyte test revealed grade 2 lymphopenia (780/μL), with a CD4^+^ count of 294/μL (normal range: 325–1,251/μL) and CD8^+^ count of 85/μL (90–775/μL). He was treated with pembrolizumab but did not survive (Patel et al., 2021). The second published case, a 56-year-old female with remittent-recurrent MS, previously treated with GA and later switched to OCR, which she received OCR for 4 years. She had 882 JC virus copies in CSF, grade 2 lymphopenia in peripheral blood (690/μL), and hypogammaglobulinemia, with IgG of 541 mg/dL (normal range: 700–1,600 mg/dL) and IgM of 24 mg/dL (40–230 mg/dL). She stabilized after treatment with pembrolizumab but was readmitted to hospital for an epileptic episode, where she died after failing to respond to anti-epileptic therapy (Puig-Casadevall et al., 2023).

NfL form part of the neuronal structure, are released into extracellular space upon cell damage or death and have therefore been proposed as markers of neuroaxonal damage (Khalil et al., 2024). NfL can be detected in CSF or serum and already serve as activity marker of MS in some centers, although their role in the follow-up of patients has yet to be established. The determination of NfL may permit the early detection of PML in at-risk patients, since increased sNfL levels have been observed up to 3 months before the diagnosis of PML in natalizumab and ozanimod-treated patients (Valentino et al., 2023; Quintanilla-Bordás et al., 2024). The study of sNfL levels may be useful to differentiate between cases of PML and relapses, given that levels in PML, which are 10-fold higher versus baseline (Dalla et al., 2019), are up to 8-fold higher than in relapses (Fissolo et al., 2021).

The present patient was diagnosed with PPMS and had received prolonged OCR treatment. Unlike the above two cases, there was no evidence of lymphopenia, no alteration in CD4^+^ or CD8^+^ T lymphocyte counts, and no reduction in immunoglobulin levels before or during the course of PML. In our prospective study of sNfL levels, these were above the normal range after PML onset. Although no data are available on pre-PML values, sNfL levels increased over the follow-up period alongside a clinical worsening and an increase in lesions on MRI scans.

This case reflects the complexity of DMT mechanisms, which can modify the immune system in ways that remain poorly understood. Clinicians should be alert to the possible onset of PML in patients receiving a DMT, even when lymphopenia is absent and immunoglobulin levels are normal. Early detection of PML may be facilitated by incorporating the determination of sNfL levels into routine clinical practice. CNS diseases other than MS, including PML, should be considered in the differential diagnosis when a rise in levels is not consistent with clinical symptoms.

## Data Availability

The original contributions presented in the study are included in the article/supplementary material, further inquiries can be directed to the corresponding author.
